# Hearing Loss: From Basic to Clinical Science

**DOI:** 10.1002/advs.202521526

**Published:** 2025-11-21

**Authors:** Renjie Chai, Hai Huang, Jing Zou

**Affiliations:** ^1^ State Key Laboratory of Digital Medical Engineering Department of Otolaryngology Head and Neck Surgery Zhongda Hospital School of Life Sciences and Technology Advanced Institute for Life and Health Jiangsu Province High‐Tech Key Laboratory for Bio‐Medical Research Southeast University Nanjing 210096 China; ^2^ Co‐Innovation Center of Neuroregeneration Nantong University Nantong 226001 China; ^3^ Southeast University Shenzhen Research Institute Shenzhen 518063 China; ^4^ Department of Neurology Aerospace Center Hospital School of Life Science Beijing Institute of Technology Beijing 100101 China; ^5^ Tulane University 6823 St. Charles Avenue New Orleans Los Angeles 70118 USA; ^6^ Department of Otorhinolaryngology‐Head and Neck Surgery Helsinki University Hospital and University of Helsinki Helsinki Finland; ^7^ Department of Otolaryngology‐Head and Neck Surgery Changhai Hospital Second Military Medical University Shanghai China

**Keywords:** hearing loss, special collection

## Abstract

Hearing loss (HL) affects over 1.5 billion people globally, with genetic factors accounting for ≈50% of congenital cases. Therefore, HL has become a global health issue, driving extensive research from basic science to clinical applications. This Special Collection includes a total of 31 papers, among which 9 are review papers, 21 are research article papers, 1 is a perspective paper, that highlight the basic mechanisms and possible protection methods of HL, the application of gene therapy for treating HL, and the clinical study and application in HL.

For hereditary HL caused by mutation of deafness genes, the collected works showcase the promising outcomes of adeno‐associated virus (AAV)‐mediated gene therapy in restoring auditory and vestibular function across various genetic forms of deafness.^[^ ^1^, ^2^, ^3^, ^4^, ^5^, ^6^ ^]^ Prof. Chai and co‐workers conduct a clinical study on AAV‐mediated OTOF gene therapy for DFNB9 deafness, validating the efficacy and safety of a GMP‐grade dual AAV‐OTOF vector in *Otof*‐mutant mice and cynomolgus macaques, and firstly demonstrating significant hearing improvement without serious adverse events in two children with *OTOF* mutations after cochlear injection (article number 2306788). Prof. Chai and co‐workers also present a comprehensive review on AAV‐mediated gene therapy for hereditary deafness, summarizing preclinical advances (vector optimization, gene strategies, animal models), non‐human primate evaluations, current clinical trials, and key challenges for clinical translation (article number 2402166). Prof. Zheng and co‐workers (article number 2410063) demonstrate long‐lasting recovery of both auditory and vestibular function following a single administration of AAV‐mediated gene replacement therapy in a novel Usher syndrome type 1C mouse model. Prof. Yuan and co‐workers (article number 2408878) show that a single administration of an optimized AAV vector named AAV‐ie‐Eh3 delivering Atp6v1b2 rescues hearing and vestibular disorders by restoring lysosomal function in hair cells in a mouse model of dominant deafness‐onychodystrophy syndrome. Prof. Sun and co‐workers (article number 2406510) report that combining AAV‐mediated Connexin 26 gene replacement with dexamethasone treatment successfully rescues hearing in a conditional Gjb2 null mouse model, proposing a novel combinatorial strategy for treating the most common form of hereditary deafness. Prof. Zheng and co‐workers (article number 2408873) identify AAVR as a key receptor for AAV transduction in sensory hair cells and demonstrate that its conditional overexpression in adult mice can restore transduction efficiency, providing a crucial insight for improving inner ear gene therapy efficacy. Prof. Pang and co‐workers (article number 2408397) comprehensively review the progress of AAV‐mediated gene therapy for inner ear diseases, covering strategies for hereditary hearing loss, hair cell regeneration for acquired loss, and preventive approaches.

The drugs for SNHL need to be delivered into the cochlea efficiently and specifically for the prevention and treatment of HL. Prof. Huan Wang and co‐workers performed a comprehensive review on the development of inner ear drug delivery systems, including the complex physiological structure that they face, types of drugs delivered, routes of administration, and forms of drug delivery carrier platforms (article number 2410568). Prof. Stankovic and co‐workers conducted a comprehensive review on the state of high‐resolution Imaging of the human inner ear, discussed the benefits and drawbacks of existing medical imaging techniques used to diagnose disorders of the human inner ear, as well as those of emerging technologies that may help overcome challenges to access, resolution, and functional detail (article number 2500556). And Prof. Gao and co‐worker established a novel polydopamine (PDA) nanogel decorated adhesive and responsive hierarchical microcarriers for controllable ALA delivery, which can effectively attenuate cisplatin‐induced ototoxicity (article number 2407637). Prof. He and co‐workers developed a multifunctional thermosensitive nanodelivery system by LR27 (a fusion peptide assembled from a targeting peptide A665 and cell‐penetrating peptide Arg8) to load and sustain the release of antioxidant Teriparatide (PTH1‐34) to alleviate HC damage and HL induced by noise exposure (article number 2408798). Prof. Tu and co‐workers developed an ultrathin, 2D sheet‐like nanoscavenger C‐TAH by modifying copper indium thiophosphate (CIPS) nanosheets with tannic acid (TA) and histone aptamers, which. Effectively binds the dsDNA of NETs/EETs, inhibits bacterial growth, and reduces reactive oxygen species (ROS) levels, leading to a depressed local inflammation in ovalbumin (OVA)‐induced OME rats (article number 2504210). Prof. Kim and co‐workers conducted a comprehensive review that delves into the intricate relationship between synaptic device technologies and neural network processing algorithms, highlighting their mutual influence on artificial intelligence capabilities (article number 2409568).

Sensorineural hearing loss (SNHL), the predominant type of HL, is primarily caused by factors such as genetics, ototoxic drugs (chemotherapeutic agents such as cisplatin, aminoglycoside antibiotics such as neomycin), noise exposure, aging, and infections. Therefore, the pathogenesis and mechanisms of HL and hearing function protection from these factors have become a key focus of hearing research. Prof. George and Prof. Ricci show that phosphatidylserine externalization does not completely describe, nor solely represent TMC scramblase activity, and that Lipid flippase/floppase activity, along with an MET‐independent scramblase, are implicated in lipid remodeling (article number 2508268). Prof. Ping Lv and co‐workers found that leucin‐rich repeat containing 8A (LRRC8A) regulates outer hair cell (OHC) volume and electromotility and is required for hearing (article number 2410477), and Prof. Dingjun Zha and co‐workers demonstrated oxidative damage related to NOX2 is highly correlated with high‐frequency OHCs vulnerability, and targeting NOX2 by ginsenoside Rg1 is identified as protective effects against neomycin‐induced hearing loss (article number 2408830). For human deafness and clinic application in HL, Prof. Huijun Yuan and co‐workers developed the Genetic Deafness Commons (GDC) by consolidating extensive genetic and genomic data from 51 public databases and the Chinese Deafness Genetics Consortium, which advances audiology research and practice by improving data accessibility and usability (article number 2408891). For noise‐induced HL (NIHL), Prof. Jian Wang and co‐workers conducted a comprehensive review on noise‐induced synaptopathy and relevant hidden hearing loss (NIS and NIHHL), with a focus on signal perception in noise and temporal processing, and also addressed the difficulty of translating animal data to humans (article number 2409322). Moreover, Prof. Shankai Yin and co‐workers proved that the anti‐inflammatory mechanism of Bilirubin, an endogenous metabolite, is targeting WNK1 to alleviate NLRP3‐mediated neuroinflammation (article number 2407349).

For protective strategies against acquired/age‐related hearing loss, several studies have also been conducted. For age‐related HL (ARHL), also known as presbycusis, Dr. Cheng Cheng and co‐workers in a review shed light on the critical role of the stria vascularis in ARHL, and explored relevant signaling pathways and therapeutic strategies for ARHL (article number 2410413). Prof. Jianming Yang and co‐workers revealed that RONIN (THAP11), through its interaction with host cell factor C1 (HCF1/HCFC1), modulated the transcriptional activity of *Tfeb*, thus protecting against D‐galactose‐induced HC senescence through autophagy activation (article number 2407880). For protection from neomycin‐induced HL, except for the former‐mentioned NOX2 (article number 2408830), Prof. Renjie Chai and co‐workers reported that Lycium barbarum glycopeptide (LBGP), a peptidoglycan isolated and purified from Lycium barbarum polysaccharides, alleviates neomycin‐induced ototoxicity by inhibiting tryptophan hydroxylase‐mediated serotonin biosynthesis (article number 2405850). For noise‐induced HL (NIHL), except for the two former mentioned articles (article number 2409322, 2408798), Prof. Sun and co‐workers conducted a comparative cochlear transcriptomics in echolocating bats and mice and found Hras as a protector against NIHL (article number 2508466). For protection from cisplatin‐induced HL (CIHL), Prof. Xia Gao and co‐workers proved that the traditional Chinese medicine monomer pinoresinol diglucoside (PDG) inhibits NCOA4‐mediated ferritinophagy by downregulating SOCS1, which reduces cisplatin‐induced ototoxicity (article number 2408777).

For the auditory circuit, regeneration and clinical translation, several investigations have also been carried out. Prof. Zeng and co‐workers (article number 2509960) present a perspective comparing cochlear implantation with the emerging field of gene therapy, highlighting the recent success of gene therapy in restoring natural hearing in patients with OTOF mutations and outlining future directions and challenges. Organoids have been used to simulate cochlear structures and replicate cochlear functions for drug development. Prof. Zhenghua Shao and co‐workers performed a comprehensive review on advancements in cochlea‐on‐a‐chip technology, including 3D cultivation of inner ear organoids and the progress in microfluidic technologies for constructing cochlea‐on‐a‐chip (article number 2406077). Prof. Qi and co‐workers (article number 2410494) review the reconstruction of the peripheral auditory circuit, discussing recent advances and future challenges in hair cell and spiral ganglion neuron regeneration utilizing biomaterials, stem cells, and gene editing technologies. Prof. Mesgarani and co‐workers presented an enhanced brain‐controlled assistive hearing system that combines auditory attention decoding (AAD) and a binaural speaker‐independent speech separation model (article number 2401379). Prof. Hao Wu and co‐workers evaluated the effectiveness of auditory brainstem implants (ABI) in pediatric patients with congenital non‐tumor hearing loss and elucidated the role of cortical plasticity in hearing and speech development (article number 2406092). Prof. Rosso and co‐workers introduced BROAD‐NESS (BROADband brain Network Estimation via Source Separation) to uncover dual‐stream mechanisms underlying predictive coding in auditory memory networks (article number 2507878). Prof. Zhang and co‐workers found that asymmetric inter‐hemisphere communication contributes to the speech acquisition of toddlers with cochlear implants (CI) (article number 2309194).

To close, this special collection focuses on the advances of HL in basic study and clinical research, including the mechanisms of HL, the protection of HCs, the gene therapy by AAV, inner ear delivery, organoids, and clinical study. We extend our appreciation to all the authors. Their valuable contributions were essential to this exciting special collection on Hearing Loss.

## Conflict of Interest

The authors declare no conflict of interest.
